# Diagnostic and Therapeutic Issues in Glioma Using Imaging Data: The Challenge of Numerical Twinning

**DOI:** 10.3390/jcm12247706

**Published:** 2023-12-15

**Authors:** Rémy Guillevin, Mathieu Naudin, Pierre Fayolle, Clément Giraud, Xavier Le Guillou, Clément Thomas, Guillaume Herpe, Alain Miranville, Christine Fernandez-Maloigne, Luc Pellerin, Carole Guillevin

**Affiliations:** 1 Department of Imaging, University Hospital Center of Poitiers, 86000 Poitiers, France; mathieu.naudin@chu-poitiers.fr (M.N.); pierre.fayolle@chu-poitiers.fr (P.F.); clement.giraud@chu-poitiers.fr (C.G.); clement.thomas@chu-poitiers.fr (C.T.); guillaume.herpe@chu-poitiers.fr (G.H.); carole.guillevin@chu-poitiers.fr (C.G.); 2 Labcom I3M, University of Poitiers, 86000 Poitiers, France; xavier.le-guillou@chu-poitiers.fr (X.L.G.); alain.miranville@math.univ-poitiers.fr (A.M.); christine.fernandez@univ-poitiers.fr (C.F.-M.); 3 DACTIM-MIS Team, Laboratoire de Mathématiques Appliquées LMA, CNRS UMR 7348, 86021 Poitiers, France; 4 Department of Genetic, University Hospital Center of Poitiers, 86000 Poitiers, France; 5 XLIM, CNRS UMR 7252, 86000 Poitiers, France; 6 IRMETIST Laboratory, INSERM U1313, University of Poitiers and University Hospital Center of Poitiers, 86000 Poitiers, France; luc.pellerin@univ-poitiers.fr

**Keywords:** numerical twin, virtual biopsy, connectome, metabolic MRI, functional MRI

## Abstract

Glial tumors represent the leading etiology of primary brain tumors. Their particularities lie in (i) their location in a highly functional organ that is difficult to access surgically, including for biopsy, and (ii) their rapid, anisotropic mode of extension, notably via the fiber bundles of the white matter, which further limits the possibilities of resection. The use of mathematical tools enables the development of numerical models representative of the oncotype, genotype, evolution, and therapeutic response of lesions. The significant development of digital technologies linked to high-resolution NMR exploration, coupled with the possibilities offered by AI, means that we can envisage the creation of digital twins of tumors and their host organs, thus reducing the use of physical sampling.

## 1. Introduction

Glial tumors, as the first etiology of primary brain tumors, are challenging both to diagnose and to treat. Yet their physical biopsy is limitative, at best partial, and sometimes impossible, due to the location of the tumor and the vulnerability of the brain. In addition, imaging, namely MRI, is the only way to assess their extensions. A standard MRI scan provides a limited morphologic aspect of a tumoral process within the brain, revealing basic information including topography, size, mass effect, extension and post-contrast increased signal, and non-quantitative parameters [1]. The rise of fluctuations of the blood oxygenation level dependent (BOLD) signal across different regions in the brain [2] has introduced a new functional dimension to brain imaging. Indeed, functional magnetic resonance imaging (fMRI) has become a widely-used tool for the investigation of cognitive processes in the human brain. It provides a new platform to explore the overall structure of local and global functional connectivity by measuring the level of resting state simultaneous activation between brain regions to detect the brain’s connectome [3,4]. This “virtual brain” also requires powerful mathematical tools such as the graph theory. Using this tool, the brain is viewed as a collection of nodes that are connected via edges [5]. The development of the connectome, or ‘wiring diagram’ of the brain, offers the potential to answer questions related to connectivity [6,7,8,9]. Then, after removing part of the network during a surgical intervention, connection re-wiring (based on brain plasticity) can be studied and predicted in silico. 

Diffusion Tensor Imaging (DTI) consists of diffusion EPI acquisition with mathematical tensor analysis. It provides an approach for tracking white matter connection patterns in 3D using white matter tractography, including multi-fiber or single fiber models [10]. These models follow coherent spatial patterns in the major eigenvectors of the diffusion tensor field [11,12,13] and can be used to complement resting state functional MRI [14]. DTI also allows quantitative measurements as mean diffusivity and fractional anisotropy that complete the glioma extension characterization. Increases in the average spacing between membrane layers will increase the diffusivity, whereas smaller spaces will lead to lower apparent diffusivities. This sensitivity makes DTI a powerful method for detecting microscopic differences in tissue properties [10]. MR scans have progressively enriched their parameters’ content with Diffusion Weighted Imaging sequences related to cell density and proliferation [15] (and perfusion weighted sequences for identifying highly aggressive areas of tumors undergoing angiogenesis [16], which can be refined by using (68Ga-PSMA-11) PET Standard Uptake Values linked with tumor vasculature [17]). 

Metabolic data are gathered from multinuclear spectroscopic and imaging MR sequences. Multiple studies, including those by our team, have shown the power of ^1^HMRS to predict (i) the ki-67 index range of value, (ii) the increase of tumor vasculature [18], (iii) the detection of 2-HG, the oncometabolite generated by the IDH 1/2 mutation, for its successful identification [19] and tumor heterogeneity assessment [20]. ^31^PMRSI provides information about cellular energetic alteration, increased membrane turnover, and tissue pH modifications [21,22,23]. Sodium imaging reveals crucial information on homeostasis and Na+/K+-ATPase function, important for maintaining an intracellularly alkaline environment in gliomas [24]. All this information allows better delineation and a more precise cutoff between low and high grade gliomas, with considerable differences in terms of prognosis. Additionally, such data can improve therapy monitoring and allows for the early detection of recurrences. Parallel to these technical developments, in the revised WHO Classification of Tumors of the Central Nervous System of 2016 (revised again in 2021), molecular biology has taken precedence over histological features [25]. Extensive molecular/metabolic knowledge coupled with mathematical models has provided the capacity to explore the in vivo human brain metabolism integrating quantitative MR parameters. More recently, there have been significant developments in digital technologies linked to the huge increase of calculation capacity, allowing an extensive and rapid analysis of high dimension data with AI algorithms. In view of the burgeoning amount of quantitative imaging information and the combination of modeling and numerical computation, it has become important to develop automated analysis tools that can incorporate integrative and simplified interpretation in a comprehensive manner into daily practice for medical doctors involved in patient management. 

To fulfill this goal, digital technologies are used to build virtual physiological organs using sets of numerical data and processes that will represent the numerical twin (see graphical abstract). Digital twin technology includes several supporting elements. The first step involves creating a detailed representation of the brain, creating an optimal support for the representation of information [26]. It brings in anatomical information for a closer representation of reality and improved guidance for reconstruction [27]. At the second step, it becomes necessary to adjust the resolutions of the data using interpolation techniques with functional data weighting. This allows doctors to work at a sufficient resolution to understand underlying biological phenomena [28]. In this process, lesion identification and segmentation are facilitated by numerous algorithms. However, it is crucial to identify the most relevant sequences to achieve segmentation [29]. Then, in a third step, it becomes possible to assess the glioma’s grade [30] and study its evolution through mathematical modeling, particularly through the trajectory of its lactate concentration [31].

In the following sections, we will further discuss these different steps, leading to the generation of a numerical twin for both the lesion (e.g., the glioma) and its host organ (the brain). This approach should provide three major advantages toward so-called 5P medicine: virtual biopsy, therapeutical simulations, and outcome prediction. 

## 2. Choosing the Parameters

From year to year, new MR sequences are proposed by research teams using different methodological procedures, thus gathering information related to pathophysiological issues [32]. The choice of each parameter must be (i) consistent with the question to be addressed, (ii) accessible with reliable, reproducible quantitative measurements, (iii) within a reasonable acquisition time for the patient, (iv) integrable into a model, and (v) not redundant with other information. In addition, key genetic and molecular pathway information needs to be captured with enough spatial resolution, as provided by high- and ultra-high field MR scanners, in the shortest acquisition time possible [33,34].

**Figure 1:**
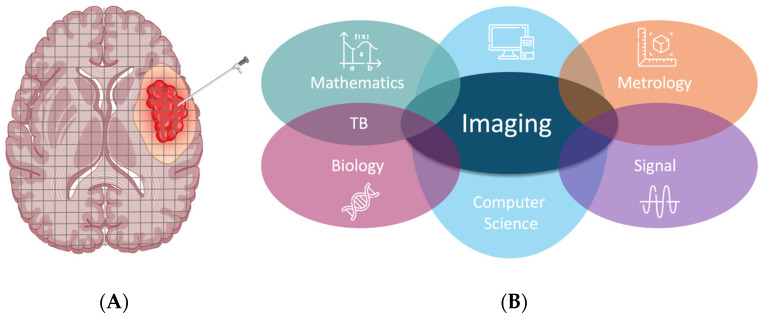
(**A**) schematic comparison between physical (needle) biopsy: focal and partial and virtual, global biopsy (grid superimposed in successive slices). (**B**) Different fields of knowledge required in a team for achieving numerical twinning for an organ in the course of its pathological process.

Once acquired, the set of parameters should be post-processed and integrated into the pipeline to be delivered as quickly as possible to the medical staff. As a consequence, the team in charge of processing this type of information should not be made up of radiologists only, but should include all the following skills: NMR methodology, metrology, signal analysis and treatment, computer science, mathematics and theoretical biology (TB) for integration into the models (Figure 1). 

If metabolic information is to be mutually consistent, the parameters for morphological analysis must be kept to a minimum. Another challenge lies in the methods of extraction, quantification, and reproducibility of the parameters chosen. Quantitative imaging can provide reliable T1 and T2 values to achieve this goal [35].

By providing anatomical and structural information about neoplasms and surrounding parenchyma, clinically-available MRI sequences enable diagnosis and grading of gliomas. Sequences such as diffusion-weighted imaging (DWI), diffusion tensor imaging (DTI), perfusion-weighted imaging (PWI), and proton MR spectroscopy (^1^H-MRS) are currently the benchmarks for the detection and assessment of a brain tumor’s oncotype. These practices combine different classifiers and machine learning [36,37]. However, the assessment of brain tumor patients, including complete extension assessment, monitoring early responses to therapy, and predicting the outcome, remains challenging [38]. More recently, a huge increase of knowledge on both the genetic profiles of these tumors and their metabolic counterparts have led to the development of multiple metabolic imaging strategies [39,40,41]. Regarding the ability of MR to investigate these aspects, multinuclear spectroscopy and imaging have been developed. Proton MR spectroscopy (^1^H-MRS), which can non-invasively assess steady-state metabolite levels, has been successfully used in clinical settings. In addition, the measurements can be performed repeatedly, thus allowing dynamic studies. An extensive mapping of the tumor and its environment can be obtained during an MRI examination, thus eliminating the uncertainty associated with the location-based choice of the physical biopsy, which is made a priori in the neurosurgical unit with the help of a neuronavigator. Furthermore, the information previously mapped can be re-inserted into the injected T1 volume used by the neuronavigator [5,42], optimizing the resection of metabolically active areas of the lesion. Tumor tissue analysis can then be performed secondarily. 

The emergence and conceptualization of the central role of lactate, not only as an escape metabolite from the Krebs cycle, but also as an energy substrate for tumor cell growth via the intratumoral lactate shuttle [43], has made it possible to use its detection and quantification (easily achieved with proton spectroscopy) to model and predict the behavior of a glioma. Thus, the detection of lactate resonance during WHO II glioma monitoring predicts (i) an increase in Ki-67 above 4% [44] and (ii) the appearance of a perfusion increment above 1.75 (rCBVmax) [18], this increment in turn being predictive of OS shortening. More recently, ^13^C MRS studies have demonstrated a drastic reduction in lactate production by IDH1 mutated gliomas that are unaffected by treatment. In vivo MRS identification of IDH1 mutation is now available in specialized centers. Yet metabolomic analysis demonstrated higher levels of 2HG and decreased glutamate levels in IDH1 mutant glioma tissue. Conventional MRS detection of glutamate and 2HG resulted in a high diagnostic accuracy [45]. Lastly, phosphorus spectroscopy data can be used to calculate the intracellular pH of the zone under study, enabling the identification of alkalinization, a marker of anaplastic transformation of a LGG [46].

Using machine learning on MRIs from glioma patients, a model was generated that predicts both IDH mutation status and 1p19q codeletion [47]. ^1^H-MRS allowed us to identify higher concentrations of Cystathionine in IDH1 patients with 1p19q codeletion [48]. The presence of a TERTp mutation in the absence of the IDH mutation is a very reliable indicator of GBM biology, while TERTp-only mutant gliomas have the worst overall survival rates. On the other hand, the presence of the TERTp mutation and 1p19q codeletion in addition to the IDH mutation indicates an oligodendroglial origin that has been reported to have a better response to chemotherapy and better outcome. As a result, an increase in 2HG and a decrease in NADPH, GSH, Glu and Gln levels have been demonstrated in IDH1 mutant gliomas.

## 3. Automatic Analysis of Tumoral Heterogeneity: The Challenge of Segmentation

All the previously mentioned genetic-metabolic information can be identified by (semi) automatic spectra analysis and should then be integrated into an AI process.

An MR-spectroscopy-based algorithm for in-depth characterization of brain lesions and prediction of a lesion’s molecular traits has been developed. Dimensional reductions of metabolic profiles demonstrated distinct patterns throughout pathologies. With a combination of a deep autoencoder and multi-layer linear discriminant models for voxel-wise prediction of the molecular profile based on MRS imaging, Diamandis et al. found specific metabolite patterns in different spatial regions [49]. Yet, choline and lactate resonance allow flagging of a contrast enhanced tumor, thus allowing metabolic prediction classification of molecular subgroups of tumors. The fingerprinting schedule consists in (i) acquisition of a sub-sampled image set and library generation via sequence parameters; (ii) comparison of the library and the signal for each voxel assignment to a tissue class (iii) creation of the resulting maps for enhanced image analysis [50]. Similar calculation of radiomic features using 11C-methionine PET can be achieved within the specific tumoral volume [51], and then integrated with MRI volumes. 

Other spatial segmentation methods for brain tumors have been developed using innovative mathematical tools. There is a morphological approach based on T2Flair volume acquisitions, using (i) a proliferation-diffusion equation δ(c)/δ(t) = *ρ*·c + ∇·(D ∇·c) [52], (ii) a diffusion tensor sequence, (iii) segmentation with extraction of the equivalent diameter of the spheroid d = ${\left(2V\right)}^{1/3}$ to obtain the equivalent diameter, which is predictive of progression-free survival [53,54,55]. However, this method, while simple to implement, requires at least one year to provide significant information, and should be used in complementarity with the previously-discussed metabolic method.

A more complex method, using a Cahn–Hilliard type equation (4th order PDE in space), integrates parametric information from MRI while, takeing into account spatial diffusion, phase separation, and aggregation phenomena (low or high cell concentrations in different parts of the tumor), thus restoring tissue heterogeneity and its temporal growth component (anisotropy) [56].

$$\frac{\partial u}{\partial t}+\alpha {∆}^{2}u-∆f\left(u\right)+\frac{ku}{k^{\prime }+u}=J\left(u,x,t\right),\alpha ,J,k,k^{\prime }>0$$

$$\frac{\partial u}{\partial v}=\frac{\partial ∆u}{\partial v}=0\;\mathrm{on}\;\Gamma$$

$${u|}_{t=0}=u0$$

This method can be implemented in an in silico model supplied with in vivo metabolic data collected during the patient’s MR examination.

## 4. Oncometabolic Representation: Dynamic Representation of Tumor Behavior 

**Figure 2:**
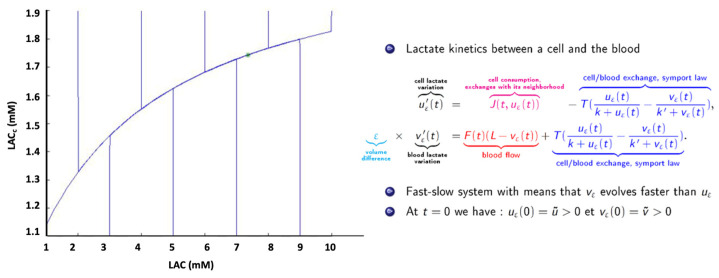
Fast-slow system. Rapid variations of [Lac] (vertical lines) around a medium value (green cross) on asymptotic trajectory, in accordance with in vivo spectroscopic quantifications.

After appropriate segmentation of the tumoral process, a dynamic estimation of key metabolites has to be performed for therapeutic monitoring and outcome prediction. An approach to estimate glioma lactate kinetics has been proposed by Perrillat et al. [57]. The two variables of the system display distinct time evolutions. Thus, the system can be studied using asymptotic and geometric analysis of slow-fast systems (Figure 2). 

The model has an associated viability domain, and generic orbits are almost parallel to the Y (LACc) axis. The generic orbits then remain in the neighborhood of the slow curve while tending toward the stationary point. As a consequence, generic orbits do not leave the viability domain. Beyond the mathematical presentation, this point means that metabolic concentrations can be analyzed in vivo using MR examination despite MR’s weak temporal resolution. 

**Figure 3:**
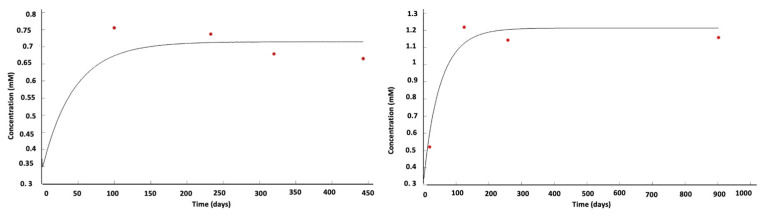
Two examples of the evolution of local lactate concentration (red dots: patient data; black curve: model simulation) in a WHO IV glioma [57]. In vivo measurements fit in silico predictions.

This approach can be extended to the various metabolites involved in tumor dynamics (e.g., glutamate) that are measurable with MRI [58,59]. Thus, by extension, the tumor is represented by a set of metabolites defining a global viability domain. When confronted with imaging data from NMR spectroscopy and perfusion, the model provides results confirming in silico simulations [57]. Then, the glioma’s process and evolution can be represented by its metabolic concentration trajectories in addition to standard imaging (Figure 3).

## 5. Other Genetic-Metabolic Issues

Analysis of the distribution of gray levels within an MRI image enables us to obtain the texture features of intra-lesional heterogeneity [60,61] which is called a Texture Analysis (TA). Based on the general assumption that a tumor’s heterogeneity should constitute a biomarker of its aggressivity, as it is correlated to the WHO grade [62], quantification of its histogram (with and without filtration) is based on the parameter standard deviation (SD), which represents the width of the histogram or degree of variation from the mean pixel value (equation shown below):

$$SD=\{\frac{1}{\left(n-1\right)}\sum_{\left(x,y\right)\in R}[a\left(x,y\right)-\bar{a}]2{\}\;}^{1/2}$$

This type of analysis can be used on different MR sequences or CT slices. However, the texture caused by necrosis, which may be important to detect during LGG transformation, may be extracted via ADC textural analysis. TA of T1 post-contrast may provide accurate quantitation of intra-lesional heterogeneity. 

Grabner et al. provided a quantitation method of local image variance of hypointensities based on 7T SWI (Susceptibility Weighted Imaging) [63], given by the formula LIV = G(X^2^) − [G(X)]^2^ (X is the pre-processed image and G represents a Gaussian low pass filtering). This method allows the detection of IDH1 mutational status by revealing significant differences in SWI-LIV values.

The fractal dimension (FD) is a non-integer number that characterizes the morphometric variability of a complex and irregular shape [64]. Two quantitative parameters can be automatically computed and correlated with each histopathological type of tumor: the volume fraction of SWI signals within tumors (signal ratio) and the morphological self-similar features (fractal dimension [FD]). 

Representing the new trend of molecular-metabolic-physiological imaging by magnetic resonance [65,66], Amide Proton Transfer-Chemical Exchange Saturation Transfer (APT CEST) may also provide critical information about the tumor response of a glioma under chemotherapy. Although this is a new technique based on a specific sequence, with only few publications in our field, CEST contrast is obtained after applying a saturation pulse at the specific resonance frequency of an exchanging proton site. The saturated spin is transferred to bulk water, and then specific molecular information can be obtained [67,68], within a so-called “negative contrast” [69]. Based on this approach, numerous low-concentration endogenous biomolecules or exogenous imaging agents with water-exchangeable chemical groups and tissue physico-chemical properties (e.g., pH) that influence the exchange rate can be detected indirectly through the bulk water signal used in MRI. Those chemical exchanges are dependent on other metabolic changes, such as the reduction in intracellular pH after treatment with TMZ, as it is documented by ^31^PMRS during treatment monitoring [70], as well as radionecrosis identification.

## 6. Building Connectomes 

### 6.1. Metabolic Connectome

Considering the above statements assessing the interdependency of multiple metabolites involved in the tumor growth process, several authors have built simulations of the impact of one variation (glucose consumption to produce lactate) on global metabolism using multiple sampling bootstrap scheme assembled metabolic brain networks with optimal parameters setup [71,72,73]. Various mathematical models can be used to simulate the interdependence of metabolic fluctuations involved in tumor growth and neuro-astrocytic function. An analytical model of glutamate/glutamine exchange dynamics integrating concentrations measured by proton spectroscopy within gliomas and surrounding tissue has been proposed by Perrillat et al. [58]. It enables us to simulate and predict the dynamic behavior of glutamate, otherwise influenced by tumor IDH status, and thus of 2 Hydroxyglutarate. It is also possible to simulate transmembrane calcium fluxes that have an impact on intracellular pH, which is measurable by phosphorus spectroscopy [74]. The same authors proposed a more global approach to the various metabolic parameters quantifiable by MRI.

It is therefore possible today to produce an in silico representation of a global biological model of a glioma, based on parameters derived from MRI. The consequences of the dynamic modifications of one metabolite upon the others can be simulated. 

### 6.2. Functional Connectome 

**Figure 4:**
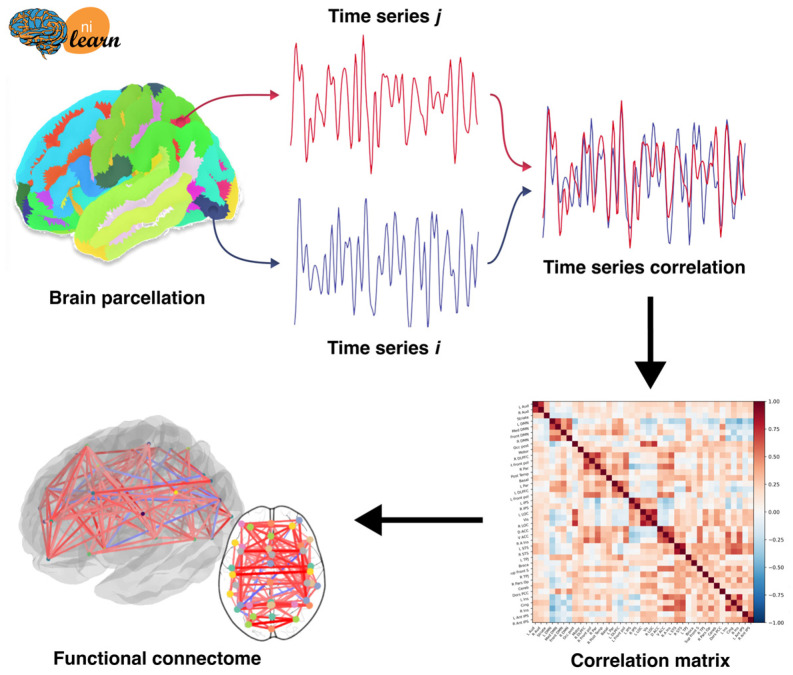
Constructing a functional map from resting fMRI data. The connectivity matrix provides temporal correlations between each possible pairs of times series. The functional network is then identified after clustering both matrix and graph representation.

Neurooncosurgery faces two major constraints that could be in opposition. It should be both as complete as possible and respectful of functional anatomy. So-called functional brain mapping has hugely increased using both pre-operative electrophysiology and MR-based BOLD acquisitions. By integrating a network-based model and localization with neuroanatomy, the brain’s connectivity is considered in a global way, i.e., holistically. Based on the graph theory, the brain is considered as a collection of nodes that are connected via edges [5,42]. The connectome analysis has revealed the brain organization—where nodes are circumscribed brain regions and edges the degree of synchronization of endogenous signals expected recovery [42]. Glioma-induced alterations of the connectome [73], including Resting State Network reorganization [75] and non-linear registration of structural data [76], may be quantified. Therefore, the graph theory measure allows the estimation of both connectivity and network topography, as it can be visualized on a virtual atlas used pre-operatively by the neurosurgeon. The virtual atlas can serve as both a pre-operative and prognostic tool for predicting functional outcomes [77] (Figure 4).

## 7. Therapeutic Simulation: Chemotherapy Modulation 

Mathematical modeling can be adapted to describe the effects of resection [78,79], chemotherapy [80,81,82], radiotherapy [83,84], or immunotherapy. Therefore it can help to plan anticancer therapy [85].

Lactate has been established as a fuel for glioma growth. Therefore, monitoring its concentration values obtained by ^1^H Magnetic Resonance Spectroscopy and integrating those values into a realistic mathematical model within slow-fast systems may be useful.

In their article, the authors used a mathematical model coupling the evolution of the tumor with the intracellular and the capillary lactate concentrations in the brain, while the tumor was being subjected to different therapeutical situations (chemotherapy and antiangiogenic treatment) for high grade gliomas. The mathematical analysis led the authors to propose applying different simulations to different therapeutical situations, including some stated by the Stupp’s protocol [86,87,88]. 

## 8. Outcome Prediction

**Figure 5:**
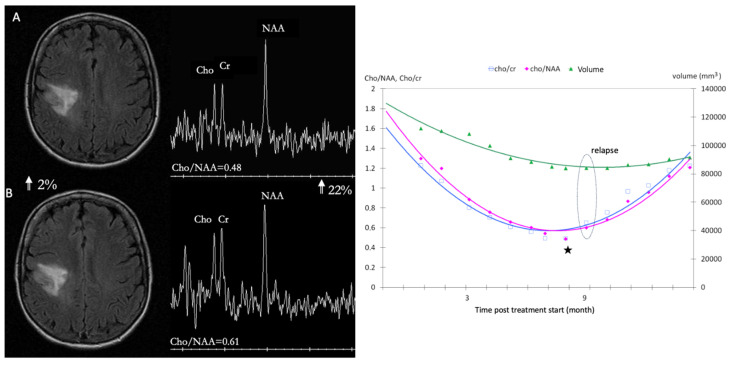
Comparison between dynamics fluctuations of metabolites ratios and volumetric measurements on LGG under TMZ treatment: stronger variations within a short time delay allowing better monitoring. (**A**) axial image FLAIR of low-grade glioma at 8 months (inflexion point ★) with associated spectrum showing a ratio Cho/NAA at 0.48, (**B**) axial image FLAIR of low-grade glioma at 9 month (circle represent the relapse) and the spectrum with a ratio Cho/NAA at 0.61. we can observe a volume augmentation of 2% and 22% for the ratio cho/NAA in one month [94].

Radiomics allows the conversion of imaging data into a high dimensional feature space using an automated data mining algorithm [89,90,91], thus overcoming single parameter analysis in patients with glioblastomas. It assesses the spatial heterogeneity of brain tumors, using clinically feasible and commonly performed T1-weighted, T2-weighted, and fluid-attenuated inversion recovery (FLAIR) MRI. They can improve the estimation of prognoses [92] and the determination of treatment response to anti-angiogenic therapy. Both perfusion WI and ^1^H-MRS have emerged as potential prognostic factors for the outcomes of glioma patients under chemotherapy. For high grade gliomas under bevacizumab, both dsc and dce markers are relevant [89,90,92,93]. For LGG under TMZ [94], the mean relative decrease of metabolic ratios, mean (D(Cho/Cr)_n_/(Cho/Cr)o), at n = 3 months is predictive of tumor response over the 14 months of follow-up (Figure 5). The mean relative change between metabolic ratios, mean ((Cho/NAA)_n_(Cho/Cr)_n_)/(Cho/NAA)_n_, at n = 4 months is predictive of tumor relapse with a significant cutoff of 0.046, a sensitivity of 60% and a specificity of 100% (*p* = 0.004), Figure 5.

PET studies using specific markers may also contribute to outcome prediction, using the Tumor to Normal Brain Uptake Ratio to characterize image geometrical properties [95].

## 9. Digital Twin: Issues

As we can define the digital twin as a mathematical object representing an organ with its pathology, here a tumor, this numerical object can be updated in real time with imaging and clinical measurements, thus leading to uncertainty accounting in the mechanistic model parameters. The aim of such a computational and mathematical tool is to smoothly integrate patient data within any given model.

Several scientific articles have focused on the evolution of gliomas, risk stratification, and patient outcomes [96,97]. However, most of these articles evaluated fixed conditions at a defined time without integrating new dimensions (temporal, multiple treatments, etc.) or data at the scale of a single patient. In their paper, Chaudhuri et al. provided patient-specific modeling and treatment planning for high grade gliomas [98]. They solved a multi-objective risk-based optimization under uncertainty problem to provide a suite of treatment plans balancing tumor control and toxicity. However, the conditions can be considered restrictive, as radiotherapy is the only therapeutic issue here.

In the realm of limitations, the focus on a single theme under specific disease stages or precise clinical criteria represents one of the elements explaining the challenges of clinical use. In this context, the portability of algorithms to solutions capable of addressing multiple pathologies is rare. The difficulty lies in establishing a complex processing flow in which multiple agents must work in concert to ensure a result based on initial findings. In clinical settings, finding elements for using AI with patients undergoing imaging examinations is challenging [96,99]. 

As the amount of data and parameters increases, AI becomes more difficult to train and requires a greater number of parameters to ensure its stability and robustness. This in turn increases computational costs and the associated costs of implementing a digital twin in clinical practice. Moreover, data heterogeneity, especially in the MRI domain, remains a persistent challenge [100]. 

Finally, intra-machine, inter-machine, and inter-manufacturer inhomogeneity present ongoing difficulties in the upstream data processing flow of AI. Indeed, artificial intelligences are most often developed on standardized and high-quality databases, conditions significantly divergent from current clinical practice, thus slowing the deployment of these agents in the clinical practice for these applications.

## 10. Conclusions and Future Directions

The above presentation has attempted to show that over the last two decades, the development of knowledge in neurobiology and genetics, in parallel with technological and methodological advances in NMR, have enabled mathematics and computer science, in particular theoretical biology and artificial intelligence, to create virtual digital representations of lesions and their host organ based on medical imaging, i.e., their digital twin. This digital representation, which is constantly being made more reliable through increased computing performance, offers a number of advantages. First, in terms of diagnosis, with the widespread use of virtual biopsies, which are global and totally innocuous. Second, the possibility of simulating surgical or chemotherapeutic treatments will be of great help and may suggest new therapeutic avenues such as targeting lactate transport. And finally, it will become predictive, with continuous monitoring enabling AI algorithms to significantly increase the accuracy of prediction of therapeutic response at the individual level, thus achieving predictive, personalized medicine based on scientific evidence. Moreover, the validation of some in silico models may lead to modifications in experimental study design, with the possibility of bypassing some preclinical testing involving animal experimentation.

## Data Availability

Not applicable.
